# Electronic cigarette smoke reduces ribosomal protein gene expression to impair protein synthesis in primary human airway epithelial cells

**DOI:** 10.1038/s41598-021-97013-z

**Published:** 2021-09-01

**Authors:** Hae-Ryung Park, Jose Vallarino, Michael O’Sullivan, Charlotte Wirth, Ronald A. Panganiban, Gabrielle Webb, Maya Shumyatcher, Blanca E. Himes, Jin-Ah Park, David C. Christiani, Joseph Allen, Quan Lu

**Affiliations:** 1 Program in Molecular and Integrative Physiological Sciences, Department of Environmental Health, Harvard T.H. Chan School of Public Health, Harvard School of Public Health, 665 Huntington Avenue, Boston, MA 02215 USA; 2 Department of Biostatistics, Epidemiology and Informatics, University of Pennsylvania, Philadelphia, PA 19104 USA; 3 Harvard T.H. Chan School of Public Health, Room 404-L401 Park Drive, Boston, MA 02215 USA

**Keywords:** Molecular biology, Diseases

## Abstract

The widespread use of electronic cigarettes (e-cig) is a serious public health concern; however, mechanisms by which e-cig impair the function of airway epithelial cells—the direct target of e-cig smoke—are not fully understood. Here we report transcriptomic changes, including decreased expression of many ribosomal genes, in airway epithelial cells in response to e-cig exposure. Using RNA-seq we identify over 200 differentially expressed genes in air–liquid interface cultured primary normal human bronchial epithelial (NHBE) exposed to e-cig smoke solution from commercial e-cig cartridges. In particular, exposure to e-cig smoke solution inhibits biological pathways involving ribosomes and protein biogenesis in NHBE cells. Consistent with this effect, expression of corresponding ribosomal proteins and subsequent protein biogenesis are reduced in the cells exposed to e-cig. Gas chromatography/mass spectrometry (GC/MS) analysis identified the presence of five flavoring chemicals designated as ‘high priority’ in regard to respiratory health, and methylglyoxal in e-cig smoke solution. Together, our findings reveal the potential detrimental effect of e-cig smoke on ribosomes and the associated protein biogenesis in airway epithelium. Our study calls for further investigation into how these changes in the airway epithelium contribute to the current epidemic of lung injuries in e-cig users.

## Introduction

The widespread use of electronic cigarettes (e-cig) is a significant public health concern. In 2017, 6.9 million (1 in 36) of adults in the US were current e-cig users^1^. In addition, more than 3.6 million youth, including 20.8% high school students and 4.9% middle school students, in the US currently used e-cig in 2018^2^. Recently, multiple studies reported a cluster of cases with acute, server respiratory distress in e-cig users^3–5^. The Centers for Disease Control and Prevention reported 2807 hospitalized lung injury cases from all 50 states, the District of Columbia, and two U.S. territories and 68 deaths in 29 states and the District of Columbia from people have a history of e-cig use or vaping as of February 18, 2020^6^. Although recent investigations have shown that vitamin E acetate, an additive in some tetrahydrocannabinol (THC)-containing e-cigarette or vaping products, is strongly linked to this outbreak, contribution of other chemicals in either THC or non-THC products still needs further investigations.

E-cig fluid contains numerous chemicals known to be toxic to the respiratory system, such as carbonyl compounds, aldehydes, fine particulate matter, metals, propylene glycol, glycerol, formaldehyde, volatile organic compounds (VOCs), and flavoring chemicals^7–17^, and bacterial endotoxins and fungal glucans^18^. The recent cases of lung injuries are characterized by non-infectious pneumonia-like symptoms with widely varying severity and types of inflammation^3–5,19^. This heterogeneity likely stems from the combinational effects of multiple chemicals and toxins in e-cig fluid^19^. Airway epithelium is the first line of defense in the lung and the direct target of chemicals in e-cig products. Although recent studies reported adverse impact of e-cig on airway epithelium^20–26^, the underlying mechanisms are not fully understood.

To fill these knowledge gaps, this study was aimed to identify transcriptomic changes and impacted biological pathways in human airway epithelium exposed to e-cig smoke solution from commercial e-cig products. We performed global transcriptomic profiling using RNA-seq in primary normal human bronchial epithelial (NHBE) cells, cultured at an air–liquid interface (ALI) to mimic the in vivo airway characteristics^27^, exposed to e-cig smoke solution obtaining by puffing commercial e-cig cartridges. We then conducted DAVID pathway analysis followed by qRT-PCR and in vitro mechanistic study to further validate RNA-seq results. We also analyzed flavoring chemicals and dicarbonyls in the e-cig smoke solution using Gas chromatography/mass spectrometry (GC/MS).

## Results

### Chemical analysis of e-cig smoke solution

**Figure 1:**
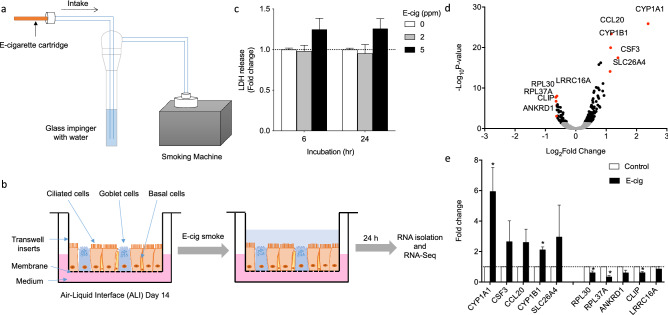
Identification of differential gene expression in NHBEs exposed to e-cigarette smoke solution by RNA-seq. (**A**) Preparation of e-cig smoke solution. (**B**) Schematic workflow of the study. (**C**) Cytotoxicity of e-cig smoke solution in NHBE cells. (**D**) Volcano plot of RNA-seq results with top 10 genes annotated in NHBE cells exposed to e-cig smoke solution for 24 h. Block dots represent gene with Padj < 0.05. Grey dots represent genes that do not meet the significance threshold. (**E**) qPCR validation of top 10 genes identified by RNA-seq with e-cig smoke solution. *, P < 0.05 compared to control. N = 3 subjects.

To generate e-cig smoke solution that closely mimics the real-world exposure to e-cig users, commercial e-cig cartridges were puffed using a smoking machine. The TE-2B smoking machine puffed twenty e-cig cartridges and the e-cig downstream smoke was collected through an impinger filled with ultrapure water (Fig. 1a). We then used GC/MS analysis to identify and quantify chemicals present in the e-cig smoke solution. Table 1 lists the compounds quantified in rank order from highest to lowest concentrations. This includes five flavoring chemicals designated as ‘high priority’ by a flavoring industry report on respiratory hazards associated with flavoring chemicals (diacetyl, benazaldehyde, acetaldehyde, propionaldehyde, and acetoin)^17^. In addition to these flavoring chemicals, we also identified the presence of methylglyoxal, an alpha dicarbonyl that is structurally related to diacetyl which can form after propylene glycol is heated in e-cig^28^. Three vanilla flavorings including ethylvanillin, 4-methoxybenzaldehyde and vanillin represented 99.7% of the compounds quantified as the vanilla e-cig flavor was used to generate the solution.

**Table 1:** Compounds Identified in e-cig smoke solution.

Compound	Concentration (ppm)	Use
Ethylvanillin	12,661.590	Flavoring
Vanillin	1384.183	Flavoring
Acetoin	198.9	Flavoring
Ethylmaltol	70.903	Flavoring
4-Methoxybenzaldehyde (4-Anisaldehyde)	50.552	Flavoring
Methylglyoxal	11.3	Non-flavoring, propylene glycol related compound
Acetaldehyde	10.7	Non-Flavoring, propylene glycol related compound
Syringol (2,6-dimethoxyphenol)	7.753	Flavoring
3-Hydroxy-2-butanone	4.804	Flavoring
Ethyl propionate	4.780	Flavoring
1-Butanol	2.227	Flavoring
2,3-Butanedione (diacetyl)*	2.1	Flavoring
Ethyl acetate	1.325	Flavoring
Piperonal	1.173	Flavoring
Benzaldehyde	0.755	Flavoring
Butyl butyryllactate	0.526	Flavoring
2,3-Dimethylpyrazine	0.524	Flavoring
Ethyl butyrate	0.521	Flavoring
2,4-Dimethylbenzaldehyde	0.471	Flavoring
Pyridine	0.295	Flavoring
Trimethylpyrazine	0.280	Flavoring
Propionaldehyde	0.126	Preservative

*The concentration of diacetyl in our in vitro study was based on the initial Mass-Spec analysis of e-cig smoke solution (89 ppm) in 2016. Due to high volatility, diacetyl in solution quickly decreased since the e-cig solution was first generated.

### Transcriptomic profiling of human bronchial epithelial cells exposed to e-cig smoke solution

To examine the effect of e-cig on human airway epithelium, we utilized air–liquid interface (ALI) cultures of primary NHBE cells. NHBE cells cultured under ALI after 14 days differentiate into a mixture of ciliated cells, goblet cells as well as some remaining basal cells, thus closely mimicking human airway epithelium in vivo^27^. Immunofluorescence staining of NHBE cells confirms the presence of ciliated and goblet cells at ALI day 14 indicating the well-differentiated state of the cells (Supplementary Fig. S1). We first exposed NHBE cells to control (DI water dissolved in culture medium, 2% v/v) or e-cig smoke solution (2% v/v, containing 2 ppm diacetyl) for 24 h (Fig. 1b). The rationale for choosing 2 ppm diacetyl was that the maximum diacetyl concentration/e-cig was about 2 ppm based on the calculation using the data from the previous study^17^. LDH (lactate dehydrogenase) assay confirmed that e-cig smoke solution did not induce significant cytotoxicity at 6 or 24 h (Fig. 1c). After 24-h incubation, total RNAs from the cells were extracted and used for RNA-Seq-based transcriptional profiling. RNA-seq libraries were constructed, each with a unique barcode that allows multiplexing. We obtained an average of ~ 18 million reads per sample and tested for differential expression of GRCh37 Ensemble-annotated genes. Following multiple testing corrections, we identified a total of 201 genes that were differentially regulated with e-cig treatment as shown in the volcano plot in Fig. 1d (Black dots, p_adj_ ≤ 0.05). A list of all statistically significant results is included in Excel File Table S1.

### qPCR validation of differentially regulated genes by e-cig smoke solution

From the list of differentially regulated genes (Excel File Table S1), we ranked the differentially regulated genes by fold change. We then selected a total of 10 genes (five most upregulated and five most downregulated genes) with each treatment for qRT-PCR validation. (Fig. 1d; Table 2). We treated ALI cultures of primary NHBE cells derived from three different donors with vehicle control or e-cig smoke solution. Out of the top 10 differentially expressed genes (Table 2), qRT-PCR verified 5 genes that were significantly changed compared to control. E-cig treatment increased expression of *CYP1B1, CYP1A1* while suppressing the expression of *RPL30, RPL37A,* and *CLIP* (Fig. 1e, p < 0.05). Because our previous study reported the transcriptional profiling of NHBE cells exposed to flavoring chemicals in e-cig products^29^, we compared the lists of significantly regulated genes exposed to diacetyl, 2,3-pentanedione, or e-cig smoke solution. We identified 24 common genes that were significantly changed with all three treatments (p_adj_ < 0.05, Supplementary Table S3). In addition, based on our previous findings showing the downregulation of cilia-involved genes by flavoring chemicals^29^, we tested the effect of e-cig smoke solution on selected cilia-related genes in NHBE cells. Consistent with previous findings, e-cig treatment downregulated expression of cilia genes in NHBE cells as shown in Supplementary Fig. S2.

**Table 2:** Differentially regulated genes in NHBE cells exposed to e-cig smoke solution.

Gene	Fold change	Padj
*CYP1A1*	5.208	7.56E−117
*CSF3*	2.6157	1.11E−20
*CCL20*	2.2513	1.31E−26
*CYP1B1*	2.2136	4.28E−24
*SLC26A4*	2.1877	1.60E−16
*RPL30*	0.6333	1.69E−08
*RPL37A*	0.6375	1.07E−07
*ANKRD1*	0.6447	0.001
*CLIP*	0.6478	1.66E−06
*LRRC16A*	0.6502	7.88E−09

### Functional annotation enrichment using DAVID

We then used the DAVID pathway analysis tool to identify ontological categories that are enriched among the differentially expressed genes induced by exposures to e-cig smoke solution. As shown in Table 3, pathways related to “SRP-dependent cotranslational protein targeting to membrane”, “Translation initiation”. “Ribosomal protein”, “Inflammatory response”, and “Cytokine”. Interestingly, the first cluster with the highest enrichment score includes pathways related to mRNA translation, ribosomes, and protein biogenesis, suggesting e-cig may perturb ribosomal functions and protein biogenesis. The full list of enriched terms is available in Excel File Table S2.

**Table 3:** Enriched terms in the gene list differentially regulated by e-cig smoke solution.

Annotation cluster 1	Enrichment score: 7.48		
Category	Term	Genes	Padj*
GOTERM_BP_DIRECT	GO:0006614 ~ SRP-dependent cotranslational protein targeting to membrane	16	9.23E−11
GOTERM_BP_DIRECT	GO:0000184 ~ nuclear-transcribed mRNA catabolic process, nonsense-mediated decay	17	1.05E−10
GOTERM_BP_DIRECT	GO:0006413 ~ translational initiation	17	6.52E−10
GOTERM_BP_DIRECT	GO:0019083 ~ viral transcription	15	5.09E−09
UP_KEYWORDS	Ribosomal protein	15	5.04E−07
GOTERM_BP_DIRECT	GO:0006412 ~ translation	17	3.74E−06
GOTERM_MF_DIRECT	GO:0,003,735 ~ structural constituent of ribosome	16	7.52E−06
KEGG_PATHWAY	hsa03010:Ribosome	15	6.05E−06
GOTERM_BP_DIRECT	GO:0006364 ~ rRNA processing	15	1.48E−05
GOTERM_CC_DIRECT	GO:0005840 ~ ribosome	13	1.18E−05
UP_KEYWORDS	Ribonucleoprotein	15	4.50E−05
GOTERM_CC_DIRECT	GO:0022625 ~ cytosolic large ribosomal subunit	8	2.69E−04
GOTERM_CC_DIRECT	GO:0022627 ~ cytosolic small ribosomal subunit	7	3.31E−04
GOTERM_CC_DIRECT	GO:0005925 ~ focal adhesion	16	3.38E−04

Annotation cluster 2	Enrichment score: 3.00		
Category	Term	Genes	Padj*
GOTERM_BP_DIRECT	GO:0006954 ~ inflammatory response	20	5.73E−06
UP_KEYWORDS	Cytokine	13	2.73E−05
INTERPRO	IPR018048:CXC chemokine, conserved site	5	0.003
INTERPRO	IPR001089:CXC chemokine	5	0.003
SMART	SM00199:SCY	6	0.003
INTERPRO	IPR001811:Chemokine interleukin-8-like domain	6	0.021
GOTERM_MF_DIRECT	GO:0045236 ~ CXCR chemokine receptor binding	4	0.020
GOTERM_MF_DIRECT	GO:0008009 ~ chemokine activity	6	0.024
KEGG_PATHWAY	hsa04062:Chemokine signaling pathway	11	0.032
GOTERM_BP_DIRECT	GO:0070098 ~ chemokine-mediated signaling pathway	6	0.074
GOTERM_BP_DIRECT	GO:0006955 ~ immune response	13	0.103

Annotation cluster 3	Enrichment score: 2.45		
Category	Term	Genes	Padj*
GOTERM_BP_DIRECT	GO:0071222 ~ cellular response to lipopolysaccharide	8	0.021

*Padj: adjusted p-values for multiple comparisons by the Benjamini Hochberg correction.

### Expression of ribosomal protein genes

**Figure 2:**
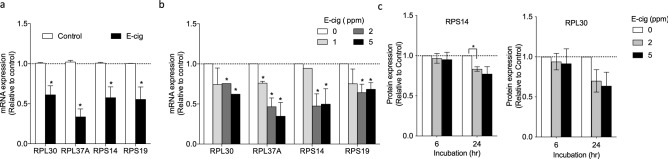
Effect of e-cig smoke solution on expression of ribosomal proteins in NHBE cells (**A**) Expression of ribosomal protein-encoding genes measured by qPCR. (**B**) Expression of ribosomal protein-encoding genes in NHBE cells at varying concentrations of e-cig solution measured by qRT-PCR. (**C**) Protein quantification of ribosomal proteins measured by Western blot. *p < 0.05 significant compared to control, N = 3 experiments.

Because the pathways involved in translation and ribosomal proteins have the lowest p-values and highest enrichment score (Table 3), we focused our follow up studies on the genes involved in ribosomal proteins. Our RNA-seq data showed that expression of 14 genes related to ribosome (*RPL30, RPL37A, RPS14, RPS19, RPL7A, RPS12, RPS11, RPL32, RPL10A, RPL 19, RPL35, RPS5, RPS21,* and *RPS 27*) was significantly down-regulated in NHBE cells treated with with e-cig treatment except that expression of *RPS29* was up-regulated (Table 4). Using qRT-PCR, we validated the down-regulation of selected genes in NHBE cells from three different donors upon e-cig treatment. Consistent with RNA-seq data, mRNA expression of *RPS14, RPS19, RPL30*, and *RPL37A* was significantly down-regulated with e-cig smoke solution (Fig. 2a, p < 0.05), suggesting that e-cig may impair ribosomal functions and protein biogenesis.

**Table 4:** Expression of ribosomal proteins from RNA seq data.

Gene	Fold change	Padj
*RPL30*	0.633	1.69E−08
*RPL37A*	0.637	1.07E−07
*RPS14*	0.656	1.84E−06
*RPS19*	0.669	2.58E−05
*RPL7A*	0.710	5.23E−05
*RPS12*	0.681	6.36E−05
*RPS11*	0.720	0.001
*RPL32*	0.703	0.001
*RPL10A*	0.721	0.001
*RPL19*	0.726	0.003
*RPS29*	1.336	0.010
*RPL35*	0.707	0.010
*RPS5*	0.759	0.020
*RPS21*	0.777	0.043
*RPL27*	0.780	0.043

To investigate the concentration-dependent effect of e-cig on ribosomal protein genes, we exposed NHBE cells to 0, 1, 2, and 5 ppm of e-cig smoke solution, then performed qPCR for *RPL30, RPL37A, RPS14,* and *RPS19*. Down-regulation of *RPL37A* was observed with e-cig smoke solution at levels as low as 1 ppm (diacetyl) (Fig. 2b, p < 0.05). Treatment with the higher concentration of e-cig solution (5 ppm diacetyl) also significantly suppressed expression of *RPS14, RPS19, RPL30*, and *RPL37A* (Fig. 2b, p < 0.05)**.** To further validate these findings, protein expression of selected targets were measured by Western blot assay. The three top hits RPL30, RPL37A, and RPS14 were selected from Table 4. RPS 14 expression showed a significant, but modest decrease at 2 ppm at 24 h. RPL 30 expression decreased at 2 and 5 ppm at 24 h, but was not significant (Fig. 2c). We suggest that the abundance of ribosomal proteins in the cells, the modest transcriptional changes, and relative insensitivity of western blot assay may have led to the difficulty in detecting protein changes. The full length blots are shown in Supplementary Fig. S4. RPL37A was not included due to non-specific binding of its antibody.

### Effect of e-cig smoke solution on ribosomal RNA transcription and protein synthesis

To further investigate the effect of e-cig on ribosomal biogenesis, we measured transcriptional levels of ribosomal RNAs including pre-rRNA transcripts and mature rRNAs. Pre-rRNA processing begins on the 47S pre-rRNA transcript, which gives rise to 45S pre-rRNA and ultimately to mature 18S, 5.8S, and 25/28S rRNAs in functional ribosomal subunits through a complex series of processing, modification, and folding steps^30–32^. Within 47S pre-rRNA transcript, the 18S, 5.8S, and 28S rRNAs are flanked by the 5′ and 3′ external transcribed spacers (ETS) and two internal transcribed spacers (ITS1 and ITS2)^30–32^. E-cig treatment at 2 ppm for 24 h decreased the expression of 47S pre-rRNA as indicated by the 5′-ETS level and increased ITS2 in NHBE cells (Supplementary Fig. S3). However, levels of ITS1 and mature rRNAs, including 28S, 18S, and 5.8S were not altered by e-cig treatment (Supplementary Fig. S3).

**Figure 3:**
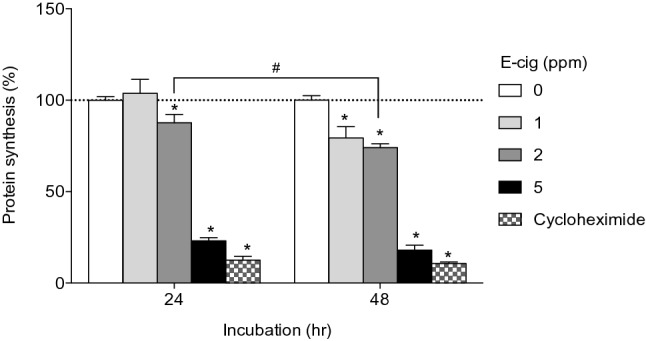
Effect of e-cig on protein synthesis in NHBE cells. *P < 0.05 significant compared to control, ^#^P < 0.05 significantly different from each other, N = 3 experiments.

To investigate the effect of e-cig on protein biogenesis, we exposed primary NHBE cells to e-cig smoke solution for 24 or 48 h and performed protein synthesis assay. As shown in Fig. 3, protein synthesis at 24 h was significantly decreased by 88% and 23% at 2 and 5 ppm, respectively, compared to control (p < 0.05). At 48 h, protein synthesis was significantly decreased by 79%, 74%, and 18% at 1, 2, and 5 ppm, respectively (p < 0.05). These data indicate that exposure to e-cig may disrupt protein biogenesis in lung epithelium.

## Discussion

The recent outbreak of e-cig or vaping associated lung injuries calls for immediate studies on their impact on human lung. Although human airway epithelium is the first line of defense in the lung and is the direct target of external chemicals, little is known about how chemicals in e-cig smoke may impair the lung epithelium and its function. The present study shows that e-cig smoke solution induces significant transcriptomic changes, including those related to ribosomal proteins and translation, affects transcription of rRNAs, and decreases new protein synthesis in primary human airway epithelial cells. The findings in this study implicate potential mechanisms by which e-cig smoking impact lung epithelium, leading to increased risks for lung diseases.

The present study performed chemical analysis on dicarnonyls and flavors in e-cig smoke solution collected from commercial e-cig products. The analysis identified “high priority” flavoring chemicals (diacetyl, benazaldehyde, acetaldehyde, propionaldehyde, and acetoin) that are associated with respiratory injuries^17^. Among them, exposure to diacetyl after heating and inhaling has been attributed to increased risk of developing bronchiolitis obliterans^33^. Our previous study found that diacetyl induce significant transcriptomic changes, including those related to ciliogenesis, and decrease the number of ciliated cells in NHBE cells^29^, implicating potential impairment of the cilia function in human airway epithelium. In addition to flavoring chemicals, we also identified the presence of methylglyoxal, an alpha dicarbonyl that is structurally related to diacetyl which can form after propylene glycol is heated in e-cigs^28^. Recent toxicological evidence found that methylglyoxal causes necrosis of respiratory epithelium and is more cytotoxic than diacetyl^34^. Mixtures of chemicals found in e-cig smoke solution may act synergistically targeting lung epithelium. Whether such cumulative influence extends to compromised cell function and lung injuries needs further investigations.

We identified that exposure to e-cig smoke solution led to significant transcriptomic changes related to protein synthesis in primary human airway epithelial cells. Specifically, expression of genes encoding ribosomal proteins, such as *RPS14, RPS19, RPL30*, and *RPL37A*, is down-regulated in NHBE cells exposed to e-cig smoke solution. Consistent with this, protein biogenesis was suppressed in NHBE cells exposed to e-cig smoke solution. In addition, we found that transcription of ribosomal RNAs was differentially regulated with e-cig smoke solution although the impact of these changes on overall protein translation needs further studies. Because ribosome biogenesis is a complex process requiring tight coordination of ribosomal RNA and ribosomal protein production^35^, our data implicates potential imbalance of rRNA and ribosome protein synthesis by e-cig exposure, thereby leading to disruption of ribosome biogenesis. Ribosome biogenesis and protein translation are finely coordinated with and essential for cell growth, proliferation, differentiation, and development. Impairment of any of these cellular processes can perturb cell growth and development^36^. It is reported that p53 play critical roles in sensing and regulating ribosomal stress and biogenesis, which in turn regulate cell cycle progression and cell growth to prevent carcinogenesis^37^. Furthermore, the balance between rRNA and ribosomal protein synthesis has been shown to control the function of p53 in mammalian cells^38^. A recent oral transcriptome analysis in by RNA-Seq revealed “cancer” as the top disease associated with dysregulated genes in e-cig users^39^. Further study on the role of p53 activation in the regulation of e-cig-mediated ribosomal stress and cell cycle regulation in relevant with carcinogenesis would be of great interest.

Regulation of translation modulates immune functions by directly affecting antigen presentation, cytokine production, as well as the survival of dendritic cells^40^. Cells need to rapidly activate the inflammatory protein synthesis in response to infection and shut it down when no longer needed^41^. Therefore, downregulation of ribosomal proteins and protein translation may potentially compromise immune functions against pathogens. On the other hand, it is reported that suppression of ribosomal function and protein synthesis activates innate immune signaling through activation of the NLRP3 inflammasome in murine bone marrow-derived macrophages^42^. In addition, the present study showed that e-cig smoke solution activates pathways related to inflammatory response and cytokines in NHBE cells (Table 3). Similarly, it is reported that e-cigs with flavorings induce inflammatory and pro-senescence responses in oral epithelial cells and periodontal fibroblasts^43^. Further study on the interaction between ribosomal proteins and immune responses and its consequences on the functions of human airway epithelium will be warranted.

Among the differentially regulated genes with e-cig smoke solution treatment, qRT-PCR validated up-regulation of *CYP1A1* and *CYP1B1*, which encode Cytochromes P450 (CYPs), key metabolizing enzymes for drugs and xenobiotics^44^. This is consistent with the previous reports showing induction of CYPs by treatment of e-cig in vitro or in vivo. For example, treatment with nicotine and e-cig fluid led to significant induction of mRNA expression for *CYP2A6, CYP2E1*, *CYP1A1* and *CYP2S1* following in an in vitro model of human brain blood barrier, the hCMEC/D3 cell line^45^. In addition, exposure to e-cig vapor increased expression of CYP1A1/2, CYP2B1/2, and CYP3A in the lungs of rats, implicating potential risks for lung cancer by bioactivation of procarcinogenes in e-cig vapor^46^. Indeed, recent studies showed that mice exposed to e-cig smoke resulted in adenocarcinomas, bladder urothelial hyperplasia, and DNA damage in mice lungs and bladders implicating e-cig smoke as a potential carcinogen in mice^47,48^. Because the present study showed the upregulation of CYP genes in human primary lung epithelial cells, further study will be warranted to investigate potential carcinogenic effects on human lungs. Interestingly, differential expression of ribosomal proteins has been also implicated in human cancer including lung cancer^49,50^. It is implicated that dysregulation of ribosomal proteins and CYPs by exposure to e-cig may have combinational/synergistic effect on cell proliferation and carcinoma in the lung.

Our previous study showed that exposure to flavoring chemicals found in e-cig such as diacetyl and 2,3-pentanedione downregulated genes related to ciliogenesis in primary NHBE cells^29^. GC/MS analysis in the present study identified diacetyl in e-cig smoke solution (Table 1). We further showed that treatment with e-cig smoke solution led to suppression of cilia-related genes in NHBE cells consistent with the effect of flavoring chemicals on these genes^29^, although this effect of e-cig smoke solution may not be solely mediated by flavoring chemicals. In addition to cilia genes, we found 24 common genes that are significantly regulated in all three groups (diacetyl, 2,3-pentanedione, and e-cig smoke solution) (Supplementary Table S3). However, different concentrations and mixture effect of various chemicals in e-cig smoke solution may contribute to the divergent responses in the expression of same genes. Full spectrum analysis of chemical composition in e-cig smoke solution will be necessary to fully understand the effect of e-cig smoke solution on lung epithelium and identify key players mediating the effect.

This is the first whole transcriptomic profiling in NHBE cells exposed to e-cig smoke solution from commercial e-cig products, however, there are a few limitations. First, mechanisms by which e-cig smoke solution impact protein synthesis is not known. Although the present study implicates that downregulation of ribosomal proteins-encoding genes may lead to retarded protein translation, it is not clear whether this phenomenon is through dysregulation of ribosomal functions or through extra-ribosomal functions that are involved in cell proliferation, differentiation, apoptosis, DNA repair, and other cellular processes^50^. We detected a modest change in protein expression of RPS 14 and RPL 30 and transcription of rRNAs, this may not fully explain the dramatic decrease of protein biosynthesis in Fig. 3A. Second, we also do not know which specific chemicals in e-cig smoke solution contribute to this outcome. Although we performed GC/MS for dicarbonyls and flavors, there is a lack of information on other chemical components such as nicotine, aldehydes, fine particulate matter, metals, propylene glycol, glycerol, formaldehyde^7–17^. Besides, since the e-cig smoke solution used in this study was collected in 2016, it may not accurately reflect the current formulations of e-cig products. Therefore, comprehensive chemical analysis on the latest e-cig formulation will be needed. Third, NHBE cells were exposed to aqueous solution of e-cig smoke that may not reflect real human exposure to e-cig. Further study using vapor exposure system will be warranted to confirm the results from this study.

Our findings reveal that e-cig smoke solution affect biological pathways related to ribosomal proteins and protein synthesis in NHBE cells. We further showed that exposure to e-cig smoke solution down-regulated expression of ribosomal proteins-encoding genes and decreased new protein synthesis from the cells. Because of the associations of increasing popularity of e-cig use among people and associations of e-cig usage and severe respiratory diseases, further mechanistic studies are warranted to evaluate the effects of e-cig on airway epithelium.

## Methods

### Preparation and analysis of e-cig smoke solution

The e-cig solution was prepared by drawing the e-cig downstream smoke through a 25 mm impinger filled with ultrapure water (Fig. 1A). The cigarettes were puffed using a TE-2B smoking machine (Teague Enterprises, Woodland, Ca). Flow rate through each cigarette was set at the minimum to ignite the e-cig. (1 to 1.5 lpm). Puffs were two seconds in duration spaced one minute apart. The e-cig cartridges using rechargeable batteries were used until no visible smoke could be seen entering the system after each puff. Twenty e-cig cartridges (Blu vivid vanilla, 2.8%) were used to make the solution. The solution was generated in a darken room over a five-date period at a rate of 4 e-cigarette cartridges per day. Ultrapure water was added to the solution each day to return the volume to 10 ml to account for solution lost to evaporation. At the initial analysis of the e-cig smoke solution detected by gas chromatography with flame ionization detector (GC-FID), the concentration of diacetyl was 89 ppm (ALS Laboratories, Cincinnati, OH). Based on this concentration, the solution was diluted into cell culture medium to treat NHBE cells in vitro. To analyze additional flavoring chemicals and dicarbonyls in the e-cig smoke solution, we performed headspace(HS)—solid-phase microextraction (SPME)—GC/MS for flavorings and gas chromatography/mass Spectrometry (GC/MS) for Di-carboynls (Labstat International, Kitchener, Ontario). The detailed methods on GC/MS are provided in Supplementary Materials.

### Cell culture and exposure

Primary normal Human Bronchial Epithelial (NHBE) cells were isolated from left over tissues after lung transplantation following the approved protocol^51^ and were received from Marsico Lung Institute/Cystic Fibrosis Center at the University of North Carolina, Chapel Hill (Chapel Hill, NC). Then NHBE cells were cultured as previously described^29,52,53^. Cells at passage 2 were transferred to microporous polyester inserts (0.4 um pore size, Transwell-Clear; Corning Costar, Corning, NY) and fed with a 1:1 mixture of BEBM and Dulbecco’s Modification of Eagle’s Media (Mediatech, Herndon, VA). Media was applied apically and basally until the cells were confluent and then basally after an air–liquid interface (ALI) was established. Cells were cultured at ALI for 14 days to promote relatively stable expression of goblet and ciliated cells before exposure to e-cig smoke solution. Mature, well-differentiated monolayers of cells were then exposed to control (ultrapure water dissolved in culture medium, water/medium: 2% v/v) or e-cig smoke solution (containing 2 ppm diacetyl, water/medium: 2% v/v) on the apical side for 6 or 24 h (n = 3 subjects, each treatment was performed in duplicate). After incubation, LDH release in the culture medium was measured for cytotoxicity assay. Total RNAs were isolated from the cells using miRNeasy kit (Qiagen) for RNA -Seq analysis.

### RNA-seq library preparation and sequencing

Libraries for sequencing were prepared as previously described^29^. Polyadenylated mRNAs were selected from total RNA samples using oligo-dT-conjugated magnetic beads on an Apollo324 automated workstation (PrepX PolyA mRNA isolation kit, Takara Bio USA). Entire poly-adenylated RNA samples were immediately converted into stranded Illumina sequencing libraries using 200 bp fragmentation and sequential adapter addition on an Apollo324 automated workstation following manufacturer’s specifications (PrepX RNA-Seq for Illumina Library kit, Takara Bio USA). Libraries were enriched and indexed using 12 cycles of amplification (LongAmp Taq 2 × MasterMix, New England BioLabs Inc.) with PCR primers which include a 6 bp index sequence to allow for multiplexing (custom oligo order from Integrated DNA Technologies). Excess PCR reagents were removed using magnetic bead-based cleanup on an Apollo324 automated workstation (PCR Clean DX beads, Aline Biosciences). Resulting libraries were assessed using a 2200 TapeStation (Agilent Technologies) and quantified by QPCR (Kapa Biosystems). Libraries were pooled and sequenced on one lane of a HiSeq 2500 high output v3 flow cell using single end, 50 bp reads (Illumina).

### RNA-seq data analysis

RNA-Seq data was analyzed as previously described^29^. Taffeta scripts (https://github.com/blancahimes/taffeta) were used to analyze the RNA-Seq data, which included trimming of adapters using trimmomatic (v.0.32)^54^ and using FastQC^55^ (v.0.11.2) to obtain overall QC metrics. Trimmed reads for each sample were aligned with STAR (v. 2.5.2b) to the reference homo sapiens build 38 UCSC file (hg38) genome obtained from the Illumina, Inc. iGenomes resource^56^. Additional QC parameters were obtained to assess whether reads were appropriately mapped. Bamtools (v.2.3.0)^57^ was used to count/summarize the number of mapped reads, including junction spanning reads. The Picard Tools (v.1.96; http://picard.sourceforge.net) RnaSeqMetrics function was used to compute the number of bases assigned to various classes of RNA, according to the hg38 refFlat file available as a UCSC Genome Table. For each sample, HTSeq (v.0.6.1) was used to quantify genes based on reads that mapped to the provided hg38 reference files^58^. The DESeq2 R package (v. 1.12.4) was used to measure significance of differentially expressed genes between the exposed (N = 4) and control (N = 4) samples and create plots of the results^59^. The reported adjusted p-values are false-discovery rate corrected to 5% according to the procedure in DESeq2 that accounts for the large number of comparisons made. An adjusted p-value < 0.05 was considered significant. The NIH Database for Annotation, Visualization and Integrated Discovery (DAVID) was used to perform gene functional annotation clustering using Homo Sapiens as background, and default options and annotation categories (Disease: OMIM_DISEASE; Functional Categories: COG_ONTOLOGY, SP_PIR_KEYWORDS, UP_SEQ_FEATURE; Gene_Ontology: GOTERM_BP_FAT, GOTERM_CC_FAT, GOTERM_MF_FAT; Pathway: BBID, BIOCARTA, KEGG_PATHWAY; Protein_Domains: INTERPRO, PIR_SUPERFAMILY, SMART)^60^.

### qPT-PCR

RNA was reverse transcribed using iScript cDNA Synthesis kit (Biorad). For rRNA measurement, cDNA was synthesized using random hexamers and SuperScript III Reverse Transcriptase (Invitrogen). The resulting cDNA was amplified using 2 × SYBR mix (Qiagen) and 1 μM of each primer in a StepOne Plus Thermocycler (Applied Biosystems) in Quantitative Reverse Transcriptase Polymerase Chain Reaction (qRT-PCR). Melt curves were checked for single-length amplification products. Fold changes were calculated using the 2−ΔΔCt method. GAPDH is the housekeeping gene used for normalization for protein coding genes. B2M was an internal control for rRNAs whose expression is not dependent on Polymerase I activity^61^. All primer sequences used in this study are listed in Supplemental Table S2.

### Western blot

Whole-cell lysates were prepared in Radioimmunoprecipitation Assay (RIPA) buffer supplemented with Protease and Phosphatase Inhibitor Cocktails (Roche, Indianapolis, Ind). Cleared lysates were heated at 70 °C for 10 min and run in SDS-PAGE gel under reducing conditions. Primary antibodies included anti RPS14 (Adcam), anti-RPL30 (Invitrogen), and anti-GAPDH (Santa Cruz Biotechnology).

### Protein synthesis assay

To test whether exposure to e-cig smoke solution impacts cells’ ability to produce new proteins, we performed protein synthesis assay using a kit from Cayman Chemical (Ann Arbor, MI, USA). This kit uses O-Propargyl-puromycin (OPP) as a probe for labeling translating polypeptide chains. OPP-labeled proteins are detected by 5-Fluorescein (FAM)-Azide. Primary NHBE cells were plated in a black, clear-bottomed 96-well plate at a density of 10,000 cells/well. After 24 h, cells were exposed to control (ultrapure water) or e-cig smoke solution (containing 1, 2, and 5 ppm of diacetyl) for 24 or 48 h. Protein synthesis was quantified following the manufacture’s protocol. Cycloheximide (500 μg/ml) was included as a positive control.

### Immunofluorescence staining

Immunofluorescence staining on ALI-cultured NHBE cells was performed as described previously with some modifications^29,62^. First, cells were fixed using 4% paraformaldehyde. Then, cells were blocked with PBS supplemented with 5% BSA and 0.2% Triton X-100 for 1 h at room temperature. Primary antibody incubation was performed overnight at 4 °C in PBS supplemented with 1% BSA and 0.2%Triton X-100 using anti-β-tubulin IV (Sigma) or anti-MUC5AC (Thermo) at 1:100 dilution. Secondary antibodies conjugated with Alexa-fluor 488 (Life Technologies) were used at 1:100 dilution. 4′-6-Diamidino-2-phenylindole, dihydrochloride was used to label the nuclear DNA and samples were mounted with Vectashield antifade mounting medium (Vector Labs, Burlingame, Calif). Confocal images were taken using Zeiss AxioObserver Z1 or Leica STP8000 and processed using ImageJ.

### Statistical analysis

Statistical analysis for in vitro studies was performed with GraphPad Prizm version 6 (La Jolla, CA 92037, USA). Data were analyzed by either t-test or one-way analysis of variance (ANOVA). If significant effects were detected, the ANOVA was followed by Tukey post-hoc comparison of means. A p < 0.05 was considered statistically different. Data were expressed as means ± SEM.

## Supplementary Information

Supplementary Information 1.

Supplementary Information 2.
