# Supplementary material for: Electronic cigarette smoke reduces ribosomal protein gene expression to impair protein synthesis in primary human airway epithelial cells
Source: Sci Rep. 2021 Sep 1;11:17517. doi: 10.1038/s41598-021-97013-z (PMC8410828; doi:10.1038/s41598-021-97013-z)
Supplement: Supplementary file 1 — Supplementary Information 1. [file 41598_2021_97013_MOESM1_ESM.docx]

**SUPPLEMENTARY MATERIALS**

**Electronic cigarette smoke reduces ribosomal gene expression to impair protein synthesis in primary human airway epithelial cells**

Hae-Ryung Park, Jose Vallarino, Michael O’Sullivan, Charlotte Wirth, Ronald A. Panganiban, Gabrielle Webb, Maya Shumyatcher, Blanca E. Himes, Jin-Ah Park, David Christiani, Joseph Allen, and Quan Lu

Corresponding Author: Quan Lu (Email: [qlu@hsph.harvard.edu](mailto:qlu@hsph.harvard.edu)) or Joseph Allen (Email: jgallen@hsph.harvard.edu)

**GC/MS Chemical analysis of e-cig smoke solution**

Liquid test sample (0.5g) was mixed with 5mL of Type I water in a 10mL glass tube. After addition of 100µL of mixed internal standard solution containing D4-acetaldehyde, D6-acetone, D5-MEK, and D6-2,3-butanedione, and 1000µL of PFBHA (20mg/mL) aqueous solution, the glass tube was capped and placed in the dark for derivatization for 24±4 hours. After derivatization with PFBHA, 5 drops of 18N H_2_SO4 were added and the PFBHA derivatives were extracted with 2mL of toluene. The toluene extract was transferred to an autosampler vial for GC/MS analysis. The PFBHA derivatives of the target carbonyls were separated on a 30m x 0.25mm i.d. x 0.25µm film thickness RTX-5ms column and then quantified by a mass spectrometer using selected ion monitoring (SIM) mode.

Semi-Quantification was completed using Related Response Factors (RRFs) derived from the LOD measurements of 61 flavor compounds potentially used in e-liquids (including potential additives used in tobacco determined by the base method) using a Root Mean Square Error (RMSE) method. This was determined using 0.25g of a solution containing propylene glycol and glycerol in a 1:1 (v/v) ratio as an artificial matrix. This matrix was spiked onto a collection pad with a series of known concentrations of flavorings to establish the calibration curves. The low level portion of the calibration curve was used to calculate the LOD without using the internal standard compensation and the high-level portion of the calibration curve is used to determine the RRF using the internal standard method.

**Supplementary Table S1. Analytical Method Specifications for HS-SPME-GC/MS**

| **SPME Parameters** | Fiber type -50/30 µm Stableflex DVB/CAR/PDMS  New Fiber Conditioning - 270 °C for 4.0 h  Used Fiber Conditioning - 270 °C for 2.0 h  Fiber Conditioning between Samples - 270 °C for 36.0 min |
| --- | --- |
| **Sample extraction parameters** | Vial -10 mL amber HS vial, screw caped with PTFE/Silicon septa  Sample- Pad or e-juice  3.0 M KClaq - 2 mL  Incubation 50 °C for 5 min, 250 rpm interval agitation, 20 sec agitation and 2 sec rest  Extraction - 50 °C for 20 min  Parameter Specification Desorption 260 °C for 5 min in GC inlet |
| **GCMS Operation Conditions** | Gas Chromatography - Agilent 7890  Column - DB WAX 30 m L × 0.25 mm ID × 0.50 µm FD  Carrier Gas - Helium (Constant Flow) at 1 mL/min  Injector Mode - Splitless  Injector Conditions - 260°C held for 60 min.  Oven Program - 40°C for 5 min. Ramped at 4°C/min to 180°C for 0 min, Ramped at 8°C/min to 240°C for 4.5 min, and Ramped at 10°C/min to 260°C for 6 min. Total run time = 60 min  Mass Spectrometer - 5975 Network Mass Selective Detector  Mass Range 35 – 400 amu  Threshold - 150  Solvent Delay- 0.25 min  Transfer Line - 260°C MS  Source Temperature - 250°C |

**Supplementary Table S2. Primer sequences**

|  | **Forward Primer (5'-3')** | **Reverse Primer (5'-3')** |
| --- | --- | --- |
| ***CC2D2A*** | AAGATTCAACAGCACCGTCTCT | GGGATGGGTCCCTTAAGAAACTT |
| ***DNAAF1*** | CCTTGACCTTTCGCACAACAA | CAAATTCAGTACACGCAAATCGG |
| ***GAPDH*** | ACAACTTTGGTATCGTGGAAGG | GCCATCACGCCACAGTTTC |
| ***PROM1*** | AGTCGGAAACTGGCAGATAGC | GGTAGTGTTGTACTGGGCCAAT |
| ***RPL 30*** | GCTGGAGTCGATCAACTCTAGG | CCAATTTCGCTTTGCCTTGTC |
| ***RPL 37A*** | CCAAACGTACCAAGAAAGTCGG | GCGTGCTGGCTGATTTCAA |
| ***RPS 14*** | AAAGGCAGACCGAGATGAATCC | TGATGTGTAGGGCGGTGATAC |
| ***RPS 19*** | AAGCTGAAAGTCCCCGAATGG | AGTTCTCATCGTAGGGAGCAAG |
| ***SPAG17*** | CAGTATGGTGTCGTGGCAAGA | GAGCATTACCACCTACAGGTTTT |
| ***B2M*** | ATGGAGGTTTGAAGATGCC | CTAAGTTGCCAGCCCTCCT |
| ***47S 5’ETS*** | CCTGCTGTTCTCTCGCGCGTCCGAG | AACGCCTGACACGCACGGCACGGAG |
| ***47S ITS1*** | GACCCCTTGGGGGGATCG | CGCGGACACCACCCCACA |
| ***47S ITS2*** | CCCGCCCCGCGGCCCGC | CGACGCGGAAGCTCGGGA |
| ***28S*** | AGAGGTAAACGGGTGGGGTC | GGGGTCGGGAGGAACGG |
| ***18S*** | GATGGTAGTCGCCGTGCC | GCCTGCTGCCTTCCTTGG |
| ***5.8S*** | ACTCGGCTCGTGCGTC | GCGACGCTCAGACAGG |

**Supplementary Table S3. Differentially regulated genes in all treatment groups (diacetyl, 2,3-pentanedione, and e-cig smoke solution).**

| **Gene** | **Diacetyl** | | **2,3-Pentanedione** | | **E- cig smoke** | |
| --- | --- | --- | --- | --- | --- | --- |
|  | **Fold Change** | **Padj** | **Fold Change** | **Padj** | **Fold Change** | **Padj** |
| ***ALDH1A3*** | 1.40 | 2.85E-06 | 1.46 | 3.85E-10 | 1.69 | 5.72E-17 |
| ***SOD2*** | 0.76 | 0.01 | 0.64 | 1.76E-12 | 1.59 | 7.54E-12 |
| ***RAB12*** | 1.37 | 1.49E-03 | 1.24 | 0.02 | 1.71 | 9.34E-11 |
| ***NDUFC1*** | 1.30 | 0.03 | 1.43 | 1.12E-04 | 1.67 | 9.56E-09 |
| ***RPL30*** | 0.79 | 0.02 | 0.80 | 0.01 | 0.63 | 1.69E-08 |
| ***CXCL1*** | 0.75 | 0.01 | 0.54 | 7.09E-26 | 1.49 | 3.64E-08 |
| ***EMC2*** | 0.77 | 0.01 | 0.65 | 0.02 | 0.66 | 3.51E-07 |
| ***SLC25A29*** | 0.76 | 3.21E-03 | 0.45 | 1.72E-27 | 0.65 | 3.29E-06 |
| ***DHRS9*** | 1.52 | 4.04E-10 | 1.60 | 3.65E-11 | 1.41 | 3.43E-06 |
| ***SERPINB4*** | 1.33 | 0.03 | 1.27 | 0.03 | 1.53 | 4.09E-06 |
| ***KRT5*** | 1.24 | 0.04 | 1.56 | 3.26E-13 | 0.70 | 5.73E-06 |
| ***PTGS2*** | 0.63 | 7.76E-08 | 0.30 | 1.15E-48 | 1.51 | 9.52E-06 |
| ***KRT15*** | 1.27 | 2.35E-03 | 1.35 | 1.04E-06 | 0.75 | 1.17E-04 |
| ***TTC9*** | 1.29 | 0.01 | 1.49 | 2.52E-08 | 1.36 | 7.54E-04 |
| ***GSR*** | 1.28 | 4.43E-03 | 1.46 | 3.83E-08 | 1.33 | 9.07E-03 |
| ***WASF2*** | 1.27 | 0.01 | 1.25 | 2.21E-03 | 1.31 | 1.18E-03 |
| ***C8orf4*** | 0.70 | 3.91E-03 | 0.43 | 2.33E-17 | 1.38 | 2.75E-03 |
| ***NFKBIA*** | 0.76 | 0.01 | 0.77 | 1.16E-03 | 1.31 | 0.01 |
| ***GCNT3*** | 1.35 | 0.01 | 1.31 | 0.01 | 1.35 | 0.01 |
| ***C15orf48*** | 0.73 | 2.03E-03 | 0.67 | 3.48E-05 | 1.31 | 0.03 |
| ***ATG16L2*** | 0.72 | 0.03 | 0.41 | 3.73E-10 | 0.72 | 0.04 |
| ***EIF1*** | 1.31 | 0.01 | 1.40 | 2.61E-06 | 1.30 | 0.04 |
| ***TNFAIP3*** | 0.72 | 3.51E-03 | 0.63 | 4.53E-07 | 1.30 | 0.05 |
| ***ANPEP*** | 0.74 | 0.01 | 0.56 | 5.50E-10 | 0.78 | 0.05 |

**Supplementary Figure S1. Immunofluorescence staining**

NHBE cells at ALI day 14 were stained for β-tubulin IV (a marker for ciliated cells) or MUC5AC (a marker for goblet cells). (A) The first panel shows nuclei visualized by DAPI staining. The second panel shows ciliated cells stained for β-tubulin IV. The third panel shows a merged image of the two panels. Representative images are shown. (B) The first panel shows nuclei visualized by DAPI staining. The second panel shows goblet cells stained for MUC5AC. The third panel shows a merged image of the two panels. Representative images are shown.

**Supplementary Figure S2. Expression of cilia-related genes in NHBE cells exposed to e-cig smoke solution.**

Primary NHBE cells were exposed to e-cig smoke solution for 24 h, then mRNA expression of *SPAG17, DNAAF1, CC2D2A, PROM1* was measured by qRT-PCR.

**Supplementary Figure S3. Effect of e-cig on rRNA transcription and processing.**

Primary NHBE cells were exposed to e-cig smoke solution for 24 h, then rRNA expression was measured by qRT-PCR.

**A**

**B**

**Supplementary Figure S4. Quantification of RPS14 and RPL30 protein expression by Western blot assay. A) RPS 14, B) RPL30**
